# Epicardial adipose tissue is related to arterial stiffness and inflammation in patients with cardiovascular disease and type 2 diabetes

**DOI:** 10.1186/s12872-018-0770-z

**Published:** 2018-02-13

**Authors:** Shaween Al-Talabany, Ify Mordi, J. Graeme Houston, Helen M. Colhoun, Jonathan R. Weir-McCall, Shona Z. Matthew, Helen C. Looker, Daniel Levin, Jill J. F. Belch, Fiona Dove, Faisel Khan, Chim C. Lang

**Affiliations:** 1 Division of Molecular and Clinical Medicine, University of Dundee, Mailbox 2, Ninewells Hospital & Medical School, Dundee, DD1 9SY UK; 2 NHS Tayside Clinical Radiology, Ninewells Hospital, Dundee, DD1 9SY UK; 3 Institute of Genetics and Molecular Medicine, University of Edinburgh, Edinburgh, EH4 2XU UK; 4 Dundee Epidemiological and Biostatistics Unit, University of Dundee, Dundee, DD1 9SY UK

**Keywords:** Epicardial adipose tissue, Pulse wave velocity, Arterial stiffness, Cardiovascular magnetic resonance, Left ventricular mass, Type 2 diabetes mellitus

## Abstract

**Background:**

Epicardial adipose tissue (EAT) is an emerging cardio-metabolic risk factor and has been shown to correlate with adverse cardiovascular (CV) outcome; however the underlying pathophysiology of this link is not well understood. The aim of this study was to evaluate the relationship between EAT and a comprehensive panel of cardiovascular risk biomarkers and pulse wave velocity (PWV) and indexed left ventricular mass (LVMI) in a cohort of patients with cardiovascular disease (CVD) and diabetes compared to controls.

**Methods:**

One hundred forty-five participants (mean age 63.9 ± 8.1 years; 61% male) were evaluated. All patients underwent cardiovascular magnetic resonance (CMR) examination and PWV. EAT measurements from CMR were performed on the 4-chamber view. Blood samples were taken and a range of CV biomarkers was evaluated.

**Results:**

EAT measurements were significantly higher in the groups with CVD, with or without T2DM compared to patients without CVD or T2DM (group 1 EAT 15.9 ± 5.5 cm^2^ vs. group 4 EAT 11.8 ± 4.1 cm^2^, *p* = 0.001; group 3 EAT 15.1 ± 4.3 cm^2^ vs. group 4 EAT 11.8 ± 4.1 cm^2^, *p* = 0.024). EAT was independently associated with IL-6 (beta 0.2, *p* = 0.019). When added to clinical variables, both EAT (beta 0.16, *p* = 0.035) and IL-6 (beta 0.26, *p* = 0.003) were independently associated with PWV. EAT was significantly associated with LVMI in a univariable analysis but not when added to significant clinical variables.

**Conclusions:**

In patients with cardio-metabolic disease, EAT was independently associated with PWV. EAT may be associated with CVD risk due to an increase in systemic vascular inflammation. Whether targeting EAT may reduce inflammation and/or cardiovascular risk should be evaluated in prospective studies.

**Electronic supplementary material:**

The online version of this article (10.1186/s12872-018-0770-z) contains supplementary material, which is available to authorized users.

## Background

The distribution of body fat appears to be important in cardiovascular disease (CVD) risk [1]. With recent advances in imaging techniques, attention has focussed on ectopic visceral fat distribution including the fat around the heart, known as epicardial adipose tissue (EAT). This interest is underpinned by population cohort studies that reported the association between EAT and adverse CV outcome [2, 3]. EAT is a metabolically active visceral and perivascular fat depot, surrounding the myocardium and coronary arteries with no separating fascia. Under pathologic circumstances, EAT can act both locally or by paracrine secretion of mediators including adipokines, inflammatory cytokines, or reactive oxidative species that can potentially adversely affect the adjacent coronary vessels and myocardium [4]. Local secretion of bioactive molecules by the EAT has been implicated in the formation of atherosclerotic plaques on the adjacent coronary arteries [5–8]. The local effects of these bioactive molecules on the neighbouring myocardium have also been linked to atrial remodelling in atrial fibrillation (AF) [9]. It is noteworthy that the effects of EAT on left ventricular remodelling such as left ventricular hypertrophy is less well studied.

Recent studies have shown that EAT is related to atherosclerotic disease in other vascular beds. In the population based Rotterdam Study, EAT was associated with multiple vessel beds and was independent of CV risk factors [10]. This observation may imply endocrine systemic effects of EAT [4]. EAT is metabolically active, secreting numerous substances associated with CVD such as TNFα, IL-6 and IL-1β [11] that may suggest a systemic effect of EAT mediated via metabolic and inflammatory pathways. Exploring this link might help to provide a better understanding of the mechanism by which EAT is associated with CVD. Tentative supportive evidence of a systemic effect of EAT is provided by the association between EAT and arterial stiffness, a known predictor of adverse CV outcome [12]. Previous studies have demonstrated an independent association between EAT and arterial stiffness in various groups of patients with CVD risk [13, 14]. However, this relationship has not been studied in patients with cardio-metabolic disease and as yet the mechanism by which EAT and arterial stiffness are linked is poorly understood. The relationship between EAT and left ventricular hypertrophy in patients with cardio-metabolic disease has also not been studied.

The aim of this study was to assess the association between EAT, metabolic and inflammatory biomarkers, peripheral arterial stiffness (as measured by PWV) and left ventricular mass (LVM) in a cohort of patients with a spectrum of cardio-metabolic disease compared to controls to provide an understanding of the underlying local and systemic pathophysiological process by which EAT might lead to adverse CVD outcome.

## Methods

### Study participants

This study was a single centre observational cross sectional sub-study of the multicentre SUMMIT (SUrrogate markers for Micro- and Macrovascular hard endpoints for Innovative diabetes Tools) study. Recruitment strategy and the imaging protocol have been described in detail previously [15]. In summary, 145 volunteers were recruited from the community, completed the imaging part of the SUMMIT study and were included for analysis in this study. These volunteers were categorised into one of four groups: Group 1 comprised of type 2 diabetes mellitus patients (T2DM) with clinical CVD including cerebrovascular disease, CAD and/or lower extremity arterial disease (LEAD); Group 2 included volunteers with T2DM and no clinical evidence of CVD; Group 3 included subjects with clinical evidence of CVD and no T2DM; and Group 4 was a group in whom there was no clinical evidence of either T2DM or CVD. To be included in either of the T2DM groups there had to be a diagnosis of T2DM made after the age of 35 years with no requirement for insulin in the 12 months following the diagnosis. Absence of T2DM in Groups 3 and 4 was determined via testing of haemoglobin A1C (HbA1C) levels, and defined as a level of less than 6.5% (48 mmol/ml). Volunteers were excluded from the study if they had a contraindication to MRI, impaired renal function (eGFR < 30 ml/min/1.73m^2^), and therapy for any chronic inflammatory disease or malignancy. The study protocols were approved by The East of Scotland Research Ethics Committee, and were conducted in accordance with the Declaration of Helsinki. All volunteers who participated in this study provided written informed consent. Informed consent was obtained from each patient and the study protocol conformed to the ethical guidelines of the 1975 Declaration of Helsinki.

### Cardiovascular magnetic resonance protocols

Cardiovascular magnetic resonance (CMR) images were acquired on a 32 RF receiver channel, 3.0 T Tesla MRI scanner (Magnetom Trio, Siemens, Erlangen, Germany). Full details of the CMR protocol and intra-observer reproducibility of LV analysis in this cohort have been as previously described in detail [15].

### Epicardial adipose tissue quantification

**Fig. 1:**
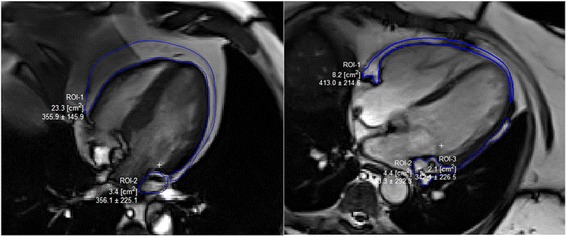
Cardiovascular Magnetic Resonance Assessment of Epicardial Adipose Tissue. EAT was assessed on CMR cine images shown in this example. A – a patient in group 1 (type 2 diabetes and overt CV disease) with EAT measuring 26.7cm^2^. B – a patient in group 4 (no diabetes or overt CV disease with EAT measuring 10.6cm^2^

EAT area was defined as the fat located between the outer wall of the myocardium and the visceral layer of the pericardium [16]. We chose a single cine four-chamber view of each patient for EAT quantification (in cm^2^) after a careful examination of the cine 4 chamber view image to outline the fat at the end-diastolic image as described in other studies [17–19]. EAT image analysis was carried out off-line using Segment (version 1.9, http://segment.heiberg.se) [20]. A representative example is shown in Fig. 1. Measurements were obtained using a TrueFISP electrocardiogram-gated cine four-chamber view during the end diastolic phase of the cardiac cycle and EAT was delineated using the freehand region of interest tool (Fig. 1). Measurement of EAT was performed with the analyser (SAT) blinded to both CVD and DM status. EAT was measured by two independent observers (SAT and JMW) in 20% of the studies (*n* = 29) selected at random to assess inter-observer variability.

### Pulse wave velocity measurements

Arterial stiffness was assessed by calculating PWV using a Sphygmocor device (Atcor Medical, West Ryde, Australia) as described previously [21]. In brief, carotid-femoral PWV (measured in m/s) was calculated as the difference in time between the R wave of the ECG and the foot of the pulse curve at the carotid and femoral arteries, divided by the proximal distance (carotid to the fossa jugulars) subtracted from the distal distance (fossa jugulars to femoral artery measured via the umbilicus). Blood pressure was measured three times via a cuff attached to the left arm and the mean of the last two measurements recorded only after the patient relaxed for 5 min.

### Biomarkers

Fifty-one biomarkers were analysed by the Proximity Extension Assay technique using the Proseek Multiplex CVD96*96 reagent kit (Olink Bioscience, Uppsala, Sweden) at the Clinical Biomarkers Facility*,* Science for Life Laboratory, Uppsala. The full list of biomarkers measured is shown in Additional file 1: Table S1.

### Statistical analysis

All analyses were performed using SPSS (version 21.0, SPSS Inc. Chicago, IL, USA). Continuous variables are shown as mean ± standard deviation. Categorical variables are reported as number with percentage in brackets. To compare the four groups, ANOVA was applied as a parametric test with post-hoc Bonferroni correction to account for multiple testing. In the investigation of associations among continuous variables, a Pearson correlation test was used for parametric data and a Spearman test for non-parametric data. Univariate and multivariable linear regressions were used for determining factors associated with EAT and PWV and LVM. Firstly, linear regression was performed to determine the association between biomarkers and EAT and PWV and LVM. Then, regression was performed to determine the independent association between clinical variables and EAT with PWV and LVM. Finally, biomarkers were added to the clinical variables and EAT to determine their association with PWV and LVM. To build the multivariable models, significant baseline clinical variables were first included. Following this, EAT and significant biomarkers were added to significant clinical variables in order to build a final multivariable model for association with PWV and LVMI. For all tests a *p* < 0.05 was considered statistically significant.

## Results

### Baseline characteristics

In total 145 participants were included in study. Full demographic details are shown in Table 1. Eighty-nine patients (61%) were male and the mean age of the whole cohort was 64.0 ± 8.1 years. Patients with DM had a significantly higher BMI, HDL cholesterol and HbA1c than those in group with neither DM nor CVD. CVD patients were significantly older than those without CVD. Patients without CVD or DM were significantly less likely to be male, have a history of hypertension, to be taking a statin and had a lower LDL-cholesterol than those in other groups. There was no significant difference in smoking prevalence or systolic blood pressure in all four groups. Patients with overt CVD were significantly more likely to be taking an ACE inhibitor and beta-blocker.

**Table 1:** Baseline Demographics, Pulse Wave Velocity and Cardiovascular Magnetic Resonance Parameters

Variable	Group 1 CVD and DM (*n* = 34)	Group 2 No CVD with DM (*n* = 54)	Group 3 CVD without DM (*n* = 28)	Group 4 No CVD or DM (*n* = 29)	*p* value
Male	28 (82.4)* ^o^	29 (53.7)	20 (71.4)	12 (41.4)	**0.003**
Age (years)	64.6 ± 6.6	62.4 ± 8.1+	67.6 ± 8.6	62.1 ± 8.0	**0.022**
BMI (kg/m2)	31.0 ± 4.2*	31.0 ± 4.6*	29.2 ± 3.6	27.9 ± 3.7	**0.007**
Diabetes duration (years)	10.3 ± 4.8	9.0 ± 6.2	N/A	N/A	0.33
Current/Ex-smoker	25 (73.5)	27 (50.0)	16 (57.1)	15 (51.7)	0.16
Hypertension	27 (79.4)*	34 (63.0)*	24 (85.7)*	7 (24.1)	**< 0.001**
Stroke	5 (14.7)*^o^	0 (0)	2 (7.1)	0 (0)	**0.008**
LEAD	9 (26.5)* ^o^	0 (0)	5 (17.9) ^o^	0 (0)	**< 0.001**
CAD	25 (73.5)*	0 (0)	22 (78.6)*^o^	0 (0)	**0.001**
Atrial Fibrillation	1 (2.9)	1 (1.9)	1 (3.6)	2 (6.9)	0.69
ACE Inhibitor	21 (61.8)*^o^	17 (30.4)	15 (53.6)*	3 (10.3)	**< 0.001**
Beta Blocker	15 (44.1)*^o^	6 (11.1)	12 (42.9)*^o^	1 (3.4)	**< 0.001**
Statin	30 (88.2)*	39 (72.2)*	22 (78.6)*	7 (24.1)	**< 0.001**
Systolic BP (mmHg)	126.3 ± 12.5	134.0 ± 14.2	130.7 ± 13.6	132.7 ± 19.1	0.13
Diastolic BP (mmHg)	73.2 ± 7.1*^o^	78.0 ± 7.4	75.5 ± 8.5	81.0 ± 9.0	**0.001**
Total Cholesterol (mmol/l)	3.7 ± 0.8*	4.0 ± 0.8*	4.0 ± 0.7*	5.1 ± 0.9	**< 0.001**
LDL Cholesterol (mmol/l)	1.7 ± 0.5*	2.0 ± 0.8*	2.0 ± 0.6*	2.8 ± 0.8	**< 0.001**
HDL Cholesterol (mmol/l)	1.1 ± 0.3*	1.3 ± 0.3*	1.3 ± 0.5	1.5 ± 0.4	**< 0.001**
Triglycerides (mmol/l)	2.1 ± 1.0+	1.7 ± 0.8	1.5 ± 0.8	1.7 ± 1.0	0.06
Serum Creatinine (μmol/l)	84.4 ± 22.6*^o^	73.3 ± 17.3	82.5 ± 19.1	70.6 ± 15.3	**0.006**
HbA1c (mmol/l)	7.5 ± 1.1*+	7.2 ± 1.1*+	5.5 ± 0.2	5.6 ± 0.2	**< 0.001**
PWV (m/s)	11.4 ± 3.0	11.1 ± 2.7	11.1 ± 2.4	9.9 ± 2.4	0.12
EAT (cm^2^)	15.9 ± 5.5*	13.5 ± 3.5	15.1 ± 4.3*	11.8 ± 4.1	**0.001**
LVMI (g/m2)	61.7 ± 9.8*	55.5 ± 10.6	59.8 ± 10.0*	52.5 ± 8.4	**0.001**
LVEF (%)	64.6 ± 10.5	65.9 ± 8.9	64.6 ± 10.6	66.0 ± 7.4	0.86
LVEDVI (ml/m^2^)	70.6 ± 12.4	67.0 ± 12.9+	75.5 ± 20.4	68.7 ± 9.9	**0.07**
LVESVI (ml/m^2^)	25.4 ± 9.8	23.2 ± 9.0	28.0 ± 16.4	23.5 ± 6.7	0.24
LGE (%)	9 (26.5)	3 (5.6)	11 (39.3)	0 (0)	**< 0.001**

*BMI* body mass index; *BP* blood pressure; *CAD* coronary artery disease, *LEAD* lower extremity arterial disease; *ACE* angiotensin converting enzyme; *ARB* angiotensin II receptor blocker; *PWV* pulse wave velocity; *LVM* left ventricular mass index; LV*EF* left ventricular ejection fraction; LV*EDV* left ventricular end diastolic volume; LV*ESV* left ventricular end systolic volume; *SV* stroke volume; *LGE* late gadolinium enhancement

Categorical data presented as number (percentage), continuous data presented as mean ± SD

*p < 0.05 vs. group 4 (no DM or CVD); ^+^*p* < 0.05 vs. group 3 (CVD without DM); ^o^*p* < 0.05 vs. group 2 (DM without CVD)

Figures in bold *p* < 0.05

### Relationship between epicardial adipose tissue, LV measurements and pulse wave velocity

PWV, EAT and LV measurements are presented in Table 1. Inter-observer variability for EAT was good (intra-class correlation coefficient of absolute agreement definition = 0.796). EAT measurements were significantly higher in the groups with CVD, with or without T2DM compared to patients without CVD or T2DM (group 1 EAT 15.9 ± 5.5 cm^2^ vs. group 4 EAT 11.8 ± 4.1 cm^2^, *p* = 0.001; group 3 EAT 15.1 ± 4.3 cm^2^ vs. group 4 EAT 11.8 ± 4.1 cm^2^, *p* = 0.024). A significant correlation was found between EAT and PWV (r = 0.28, *p* = 0.002). LVMI was also significantly higher in group 1 compared to group 4 (group 1 LVMI 61.7 ± 9.8 g/m^2^ vs. group 4 LVMI 52.5 ± 8.4 g/m^2^, *p* = 0.002). There was no significant difference in PWV amongst any of the four groups.

### Relationship between epicardial adipose tissue and blood biomarkers

The full list of 51 CV biomarkers evaluated is reported in Additional file 1: Table S1. Of these biomarkers, we found two that were significantly correlated with both EAT and PWV in univariate analysis (CD40L and IL-6). There were no biomarkers that were significantly correlated with both EAT and LVM. In multivariable linear regression analysis of biomarkers, both IL-6 and CD40L remained significantly associated with PWV (IL-6: beta 0.26, *p* = 0.003) (CD40L: beta − 0.21 *p* = 0.014) (Table 2). In a univariate regression analysis, IL6 and CD40L were significantly associated with EAT (IL6: beta 0.2 *p* = 0.019; CD40L: beta − 0.21 *p* = 0.01) (Additional file 1: Figure S1). IL-6 was significantly higher in Group 1 compared to group 4 (group 1–5.9 ± 1.3 pg/ml group 4–4.8 ± 0.7 pg/ml *p* < 0.001).

**Table 2:** Multivariable Linear Regression of PWV and Biomarkers

		PWV (m/s)		
	β	95% CI	β (S)	*p* value
CD40L	−0.5	−0.90- -0.10	− 0.21	**0.014**
IL-6	0.67	0.23–0.1.12	0.26	**0.003**

*PWV* pulse wave velocity; *CD40L* CD40 ligand; *IL-6* interleukin-6

All biomarkers significantly associated with EAT and PWV in univariate analysis (Additional file 1: Table S1) were included in multivariable analysis

Figures in bold *p* < 0.05

### Factors associated with pulse wave velocity

In univariate analysis for association with PWV we found that age, systolic blood pressure, history of smoking, lower extremity arterial disease, AF and EAT were all significantly associated with PWV (Additional file 1: Table S2). In a model with only significant clinical variables included, age (beta 0.32 *p* < 0.001), systolic BP (beta 0.34, *p* < 0.001) and lower extremity arterial disease (LEAD) (beta 0.17 *p* = 0.025) remained significantly associated with PWV (Table 3). When added to clinical variables, EAT remained associated with PWV (beta 0.12 *p* = 0.026). Finally, when IL-6 and CD40L were added to the model both EAT (beta 0.16 *p* = 0.035) and IL-6 (beta 0.20 *p* = 0.005) remained independently associated with PWV in addition to age and systolic blood pressure however CD40L was not. The stepwise additions of both EAT and IL-6 added significant independent power to model association with PWV.

**Table 3:** Multivariable associations with pulse wave velocity

	Clinical				Clinical + EAT				Clinical + EAT + Biomarkers			
	β	95% CI	β (S)	*p* value	β	95% CI	β (S)	*p* value	β	95% CI	β (S)	*p* value
Age	0.11	0.07–0.16	0.36	**< 0.001**	0.10	0.06–0.15	0.32	**< 0.001**	0.10	0.05–0.15	0.31	**< 0.001**
Systolic BP	0.06	0.04–0.09	0.37	**< 0.001**	0.07	0.04–0.09	0.37	**< 0.001**	0.07	0.04–0.09	0.39	**< 0.001**
LEAD	1.60	0.21–2.98	0.17	**0.025**	1.47	0.11–2.84	0.15	**0.035**	1.30	−0.04-2.65	0.14	0.06
Atrial fibrillation	2.03	−0.11 -4.16	0.14	0.06								
EAT					0.10	0.01–0.19	0.17	**0.026**	0.10	0.01–0.19	0.16	**0.03**
IL-6									0.53	0.16–0.90	0.20	**0.005**
CD40L									−0.03	−0.37-0.31	−0.01	0.86
Chi-Square	0.613				0.634*				0.658^			

*BP* blood pressure; *LEAD* lower extremity arterial disease; *EAT* epicardial adipose tissue; *IL-6* interleukin 6, *CD40L* CD40 ligand, (S) standardized

*p 0.026 vs. *clinical; ^p* = 0.011 vs. clinical + EAT

Figures in bold *p* < 0.05

### Factors associated with left ventricular mass

In univariable analysis there were several significant clinical variables associated with LVMI, (Additional file 1: Table S2). EAT was also significantly associated with LVMI in univariate analysis (beta 0.33, *p* = 0.002). In multivariable analysis, only male gender and history of hypertension remained significantly associated with LVMI (Additional file 1: Table S3). When added to significant clinical variables EAT was no longer significantly associated with LVMI (beta 0.07, *p* = 0.33).

## Discussion

In this study we identified several novel findings. First, we have shown that patients with overt cardio-metabolic disease have significantly more EAT than those without. Second, we have shown that EAT is significantly associated with PWV, a marker of arterial stiffness that is linked to adverse CVD outcome. We did not, however, find any significant association between EAT and LVM. Finally, we have shown, to the best of our knowledge, for the first time, a clear association between EAT and IL-6, a biomarker associated with vascular inflammation, and that both EAT and IL-6 are significantly independently associated with PWV even when baseline clinical characteristics are accounted for and have incremental association PWV when added to clinical variables.

Arterial stiffness measured by PWV has been associated with an increased risk of CVD in a wide range of patients and has been found to be an independent predictor of major adverse CV events [12]. Furthermore, it has also been shown to have incremental additional prognostic value when used in combination with traditional risk predictors such as blood pressure and cholesterol [22]. Our finding that EAT is a significantly independently associated with arterial stiffness as measured by PWV is consistent with previous studies that showed that EAT, measured by echocardiography, was independently associated with endothelial function measured by PWV [23]. In a study of 100 patients with biopsy-proven non-alcoholic fatty liver disease (NAFLD), a condition associated with inflammation and atherosclerosis, the authors found that epicardial adipose thickness was significantly higher NAFLD patients compared to controls and was a significant independent predictor of PWV [24]. Our study extends the results of this study to patients with cardio-metabolic disease by assessing patients with CMR (the gold-standard). In our study, the availability of a comprehensive panel of biomarkers may help provide a mechanistic understanding for these observations.

Prior studies have demonstrated that inflammation, oxidative stress, and visceral fat volume predispose to CVD [1, 25]. There have been several studies examining the link between pericardial adipose and CVD. In a study of over 6000 patients from the MESA cohort the authors found that pericardial adipose volume had a significant positive association with IL-6, and also had a borderline significance for CVD outcomes after adjustment for traditional risk factors [26]. More specifically, pericardial adipose also predicted incident coronary heart disease in a subset of these patients [27]. Finally, in a large cohort of over 1000 patients from the Framingham study, Tadros et al. reported an association between pericardial fat and inflammatory biomarkers including IL-6 [28].

More recent work has suggested that EAT is associated with systemic inflammation. A meta-analysis of 40 gene expression studies on EAT suggested that EAT might well mediate its effects on CVD through IL6 [29]. These observations lend support to the increasing recognition that EAT is a metabolically active endocrine organ and a source of pro-inflammatory adipokines that have significant impact on remote vascular tissues [30]. The findings of our study lend support to this. In our study IL-6, an inflammation mediator, was strongly associated with both EAT and with PWV. It is noteworthy that IL-6 is capable of inducing vascular smooth muscle proliferation that can lead to endothelial dysfunction and arterial stiffness [31]. In our study, we observed an incremental independent association of both EAT and IL-6 with PWV shown, for the first time, in our study. Indeed, further supporting our inflammatory theory is the association with CD40L, although this did not remain significant when included in the multivariable model. CD40L has also been associated with cardio-metabolic disease and is known to be released by adipose tissue [32, 33]. Obviously, any indication that the observed relationship between EAT and PWV are due to the effects of IL-6 remains purely speculative and cannot be inferred directly from this observational study. Additionally, the fact that EAT was still independently associated with PWV even when IL-6 was added to the model might suggest that there may be other pathways which need to be further elucidated by which EAT is associated with increased PWV.

In our study we found that although EAT was associated with LVM in a univariate analysis, it was not significant in multivariable analysis. Prior studies using echocardiography have reported differing results regarding the relationship between EAT and LVM. Fosshaug et al. evaluated 60 heart failure patients and found that EAT was related to LVM [34]. These results were replicated in a more recent study using PET-CT by Bakkum et al. in over 200 obese patients without significant CAD [35]. Both of these studies contrast the study by Gates et al. in 119 healthy males suggesting that overall body fat was linked to LVM rather than EAT [36]. Our study differs from all of these studies by using CMR, the gold-standard for assessment of LVMI. Additionally, our study is also, to the best of our knowledge, the first to include patients with CVD and T2D disease rather than just healthy volunteers. Because of this, there may however have been confounding of LVM based on medication use such as ACE inhibitors. It may however be the case that LVM is not influenced by local adipose but rather by overall adiposity and BMI. A large study from the Framingham cohort found that while pericardial adipose was associated with LVM in univariable analysis, this association was no longer significant once adjusted for body weight or visceral adipose [37].

### Limitations

As our findings come from a single-center study, these findings require confirmation within a larger multi-center setting. Due to the nature of recruitment of these patients in which the full biomarker panel was only available in a single centre, the number of patients for analysis is relatively small, although this is one of the largest papers to examine EAT, PWV and such a comprehensive panel of biomarkers using CMR. Additionally as a cross-sectional study causality cannot be proven and only associations can be made. Although we feel that our selection of 51 biomarkers is both pragmatic and broadly covers most pathophysiologcally important biomarkers, we cannot completely exclude the possibility that there may be other biomarkers which could be important, for example adiponectin. Finally, we only performed measurement of EAT in the 4-chamber view. Theoretically 3-dimensional measurement of EAT may have provided different results, however we wished to perform a protocol that was simple to utilise and not too time-consuming for practicing clinicians. However, measurement of adipose in the 4-chamber view has shown excellent correlation with 3-dimensional quantification in several other studies [17–19]. Despite these limitations, we believe our findings are of potential importance and can be seen as hypothesis-generating.

## Conclusions

EAT is gaining prominence as a non-invasive biomarker which might be associated with adverse CV outcome, however the mechanism by which it does so remains unclear. We performed a study in patients in both controls and patients with type 2 diabetes and/or overt cardiovascular disease evaluating EAT measured by CMR and examined its relations with established CV biomarkers, arterial stiffness measured by PWV and left ventricular mass. EAT was significantly increased in patients with overt cardio-metabolic disease compared to those without and was independently associated with arterial stiffness measured by pulse wave velocity. EAT was significantly associated with increased markers of systemic inflammation (IL-6) and both EAT and IL-6 were significantly associated with PWV independent of clinical variables, suggesting potential inflammatory pathophysiological link. Additionally, both EAT and IL-6 had significant incremental association with PWV. EAT may be associated with systemic inflammation and could be a therapeutic target in cardio-metabolic disease.

## Additional file

Additional file 1: **Table S1.** Correlations between cardiovascular biomarkers and epicardial adipose tissue (EAT), pulse wave velocity (PWV) and left ventricular mass index (LVMI). **Table S2.** Univariable Linear Regression of Clinical Variables and EAT with Pulse Wave Velocity and LV Mass Index. **Table S3.** Multivariable Associations with LV Mass Index. **Figure S1.** Correlations between EAT, IL-6 and CD40L. (DOCX 88 kb)

## Abbreviations

AF: Atrial fibrillation
CVD: Cardiovascular disease
EAT: Epicardial adipose tissue
LEAD: Lower extremity arterial disease
LV: Left ventricular
LVM: Left ventricular mass
LVMI: Left ventricular mass index
PWV: Pulse wave velocity
T2DM: Type 2 diabetes mellitus
