# Supplementary material for: Epicardial adipose tissue is related to arterial stiffness and inflammation in patients with cardiovascular disease and type 2 diabetes
Source: BMC Cardiovasc Disord. 2018 Feb 13;18:31. doi: 10.1186/s12872-018-0770-z (PMC5809843; doi:10.1186/s12872-018-0770-z)

Table S1. Correlations between cardiovascular biomarkers and epicardial adipose tissue (EAT), pulse wave velocity (PWV) and left ventricular mass index (LVMI)

| **Biomarkers** | **EAT (cm²)** | | **PWV (m/s)** | | **LVMI (g/m²)** | |
| --- | --- | --- | --- | --- | --- | --- |
|  | **r** | **p value** | **r** | **p value** | **r** | **p value** |
| **IL-8**  Interleukin-8 | 0.004 | 0.97 | 0.07 | 0.42 | -0.01 | 0.95 |
| **CD40L**  CD40 ligand | -0.021 | **0.01** | -0.2 | **0.03** | -0.05 | 0.57 |
| **GDF15**  Growth/differentiation factor-15 | 0.12 | 0.15 | 0.38 | **<0.001** | 0.16 | 0.06 |
| **SELE**  selectin | 0.6 | 0.55 | 0.14 | 0.13 | 0.03 | 0.72 |
| **OPG**  Osteoprotegerin | -0.08 | 0.36 | 0.15 | 0.09 | -0.12 | 0.16 |
| **IL-1ra**  Interleukin-1Ra | 0.19 | **0.02** | 0.15 | 0.09 | 0.03 | 0.74 |
| **IL-6**  Interleukin-6 | 0.2 | **0.02** | 0.25 | **0.01** | 0.15 | 0.80 |
| **MCP1**  Monocyte chemoattractant protein-1 | 0.07 | 0.41 | 0.16 | 0.07 | -0.05 | 0.52 |
| **PAR1**  protease-activated receptors-1 | -0.09 | 0.30 | 0.02 | 0.79 | -0.09 | 0.31 |
| **TRAIL**  Apo2 ligand | 0.01 | 0.91 | 0.06 | 0.51 | -0.03 | 0.73 |
| **TNFR1**  tumour necrosis factor receptor 2 | 0.13 | 0.11 | 0.25 | **0.01** | 0.07 | 0.43 |
| **IL-27A**  Interleukin-27A | 0.02 | 0.78 | 0.18 | **0.04** | 0.003 | 0.97 |
| **CSF1**  colony stimulating factor 1 | 0.02 | 0.82 | 0.11 | 0.23 | -0.04 | 0.66 |
| **CXCL1**  chemokine (C-X-C motif) ligand 1 | -0.11 | 0.20 | -0.2 | **0.03** | -0.02 | 0.83 |
| **LOX1**  Lectin-like oxidized LDL receptor 1 | 0.09 | 0.28 | 0.14 | 0.11 | 0.11 | 0.21 |
| **TRAILR2**  Apo2 ligand R2 | 0.07 | 0.39 | 0.19 | **0.04** | 0.07 | 0.41 |
| **IL-18**  Interleukin-18 | 0.1 | 0.24 | 0.07 | 0.43 | -0.01 | 0.93 |
| **IL-6RA**  Interleukin-6RA | 0.005 | 0.95 | -0.01 | 0.90 | -0.16 | 0.05 |
| **TNFR2**  tumour necrosis factor receptor 2 | 0.09 | 0.30 | 0.22 | **0.02** | 0.04 | 0.60 |
| **MMP3**  matrix metalloproteinase-3 | 0.04 | 0.67 | 0.08 | 0.37 | 0.25 | **0.003** |
| **TNFSF14**  tumor necrosis factor superfamily member 14 | -0.1 | 0.24 | -0.003 | 0.98 | 0.09 | 0.29 |
| **MPO**  Myeloperoxidase | -0.01 | 0.88 | 0.07 | 0.45 | -0.47 | 0.64 |
| **MMP1**  Matrix metalloproteinases 1 | 0.004 | 0.96 | 0.15 | 0.11 | 0.04 | 0.68 |
| **FAS**  Tumor necrosis factor receptor superfamily member 6 | 0.17 | 0.05 | 0.3 | 0.001 | -0.03 | 0.73 |
| **PTX3**  Pentraxin-related protein | 0.002 | 0.99 | 0.22 | **0.04** | 0.50 | 0.62 |
| **REN**  Renin | 0.12 | 0.16 | 0.08 | 0.40 | 0.29 | **<0.001** |
| **CHI3L1**  Chitinase-3-like protein 1 | 0.13 | 0.12 | 0.19 | **0.03** | -0.02 | 0.78 |
| **ST2**  ST2 protein | -0.02 | 0.79 | 0.05 | 0.58 | 0.16 | 0.06 |
| **TRANCE**  Tumor necrosis factor related activation-induced cytokine | 0.1 | 0.25 | -0.02 | 0.79 | 0.03 | 0.70 |
| **PSGL1**  P-selectin glycoprotein ligand-1 | -0.02 | 0.83 | 0.1 | 0.34 | -0.16 | 0.10 |
| **IL-16**  Interleukin-16 | -0.02 | 0.84 | 0.09 | 0.35 | -0.02 | 0.78 |
| **MMP10**  Matrix metalloproteinase 10 | 0.05 | 0.53 | 0.19 | **0.03** | 0.07 | 0.40 |
| **CCL4**  C-C motif chemokine-4 | 0.08 | 0.37 | 0.18 | 0.05 | 0.06 | 0.50 |
| **RAGE**  Receptor of Advanced glycation end products | -0.05 | 0.57 | -0.07 | 0.41 | -0.06 | 0.45 |
| **CCL3**  C-C motif chemokine-3 | 0.03 | 0.74 | 0.02 | 0.80 | -0.09 | 0.30 |
| **MMP7**  Matrix metalloproteinase-7 | 0.03 | 0.73 | 0.06 | 0.50 | 0.06 | 0.45 |
| **CXCL6**  Chemokine (C-X-C motif) ligand-6 | -0.04 | 0.63 | 0.06 | 0.54 | -0.09 | 0.30 |
| **CXCL16**  Chemokine (C-X-C motif) ligand-16 | -0.004 | 0.96 | 0.19 | **0.04** | -0.09 | 0.31 |
| **ENRAGE**  Receptor of Advanced glycation end products ligand | 0.04 | 0.67 | -0.05 | 0.60 | 0.10 | 0.30 |
| **CD40**  CD40 receptor | -0.15 | 0.08 | -0.07 | 0.45 | -0.05 | 0.52 |
| **HB-EGF**  Heparin-binding EGF-like growth factor | -0.15 | 0.08 | -0.06 | 0.52 | -0.01 | 0.91 |
| **ESM1**  Endothelial cell-specific molecule 1 | -0.17 | 0.05 | -0.06 | 0.53 | -0.13 | 0.11 |
| **MMP12**  Matrix metalloproteinase-12 | 0.08 | 0.34 | 0.25 | **0.01** | 0.18 | **0.03** |
| **CTSL1**  Cathepsin L1 | 0.02 | 0.83 | 0.14 | 0.13 | 0.08 | 0.36 |
| **CX3CL1**  chemokine (C-X3-C motif) ligand-1 | -0.07 | 0.43 | 0.003 | 0.97 | -0.05 | 0.60 |
| **BNP**  B-type natriuretic peptide | -0.06 | 0.55 | 0.08 | 0.52 | 0.23 | **0.03** |
| **CCL20**  C-C motif chemokine-20 | 0.02 | 0.85 | 0.06 | 0.49 | -0.07 | 0.42 |
| **NEMO**  NF-kappa-B essential modulator | -0.15 | 0.09 | -0.15 | 0.1 | 0.04 | 0.64 |
| **FS**  Follistatin | -0.05 | 0.56 | 0.07 | 0.46 | -0.08 | 0.36 |
| **PECAM1**  Platelet endothelial cell adhesion molecule 1 | -0.14 | 0.09 | 0.02 | 0.83 | -0.17 | **0.04** |
| **NT-pro-BNP**  N-terminal pro-B-type natriuretic peptide | -0.02 | 0.84 | 0.24 | 0.01 | 0.24 | **0.007** |

Table S2. Univariable Linear Regression of Clinical Variables and EAT with Pulse Wave Velocity and LV Mass Index.

|  |  | **PWV (m/s)** | | |  | **LVMI (g/m2)** | | |
| --- | --- | --- | --- | --- | --- | --- | --- | --- |
|  | **β** | **95% CI** | **β (S)** | **p value** | **β** | **95% CI** | **β (S)** | **p value** |
| **Age (years)** | 0.15 | 0.10 - 0.20 | 0.46 | **<0.001** | 0.01 | -0.21 – 0.22 | 0.004 | 0.97 |
| **Male** | -0.50 | -1.46 - 0.46 | -0.1 | 0.31 | -11.83 | -14.76 - -8.91 | -0.66 | **<0.001** |
| **BMI (kg/m²)** | 0.07 | -0.05 – 0.18 | 0.10 | 0.27 | 0.32 | -0.07 – 0.72 | 0.14 | 0.11 |
| **Systolic BP (mmHg)** | 0.08 | 0.05 - 0.11 | 0.46 | **<0.001** | 0.06 | -0.06 – 0.17 | 0.08 | 0.32 |
| **Diabetes** | -0.81 | -1.75 – 0.14 | - 0.15 | 0.09 | -1.72 | -5.22 – 1.78 | -0.08 | 0.33 |
| **Creatinine (µmol/l)** | 0.02 | -0.004 – 0.45 | 0.15 | 0.11 | 0.16 | 0.08 – 0.25 | 0.30 | **<0.001** |
| **Total Cholesterol** | -0.26 | -0.75 – 0.24 | -0.09 | 0.31 | -3.17 | -4.94 - -1.40 | -0.29 | **<0.001** |
| **LDL-Cholesterol (mmol/l)** | -0.44 | -1.05 – 0.17 | -0.14 | 0.16 | -2.77 | -5.00 - -0.50 | -0.21 | **0.017** |
| **HDL-Cholesterol (mmol/l)** | -0.93 | -2.11 – 0.25 | -0.14 | 0.12 | -6.92 | -11.11 - -2.74 | -0.27 | **0.001** |
| **Triglycerides (mmol/l)** | 0.33 | -0.20 – 0.86 | 0.11 | 0.22 | 0.38 | -1.52 – 2.28 | 0.03 | 0.69 |
| **Smoker/Ex-smoker** | 0.94 | -0.001 – 1.87 | 0.18 | **0.05** | 1.74 | -1.72 – 5.20 | 0.08 | 0.32 |
| **Hypertension** | 0.59 | -0.37 – 1.54 | 0.11 | 0.23 | 6.71 | 3.31 – 10.11 | 0.31 | **<0.001** |
| **CAD** | 0.71 | -0.31 – 1.72 | 0.12 | 0.17 | 5.39 | 1.79 – 8.98 | 0.24 | **0.004** |
| **LEAD** | 2.16 | 0.49 – 3.83 | 0.23 | **0.012** | 7.02 | 1.17 – 12.88 | 0.17 | **0.019** |
| **Stroke** | -0.12 | -2.53 – 2.28 | -0.01 | 0.92 | 7.15 | -0.74 – 15.04 | 0.15 | 0.08 |
| **Atrial Fibrillation** | 3.49 | 0.88 – 6.09 | 0.23 | **0.009** | -4.98 | -14.31 – 4.35 | -0.09 | 0.29 |
| **ACE Inhibitor** | 0.18 | -.079 – 1.15 | 0.04 | 0.70 | 5.18 | 1.76 – 8.60 | 0.24 | **0.004** |
| **Beta Blocker** | 0.91 | 3.17 – 10.91 | 0.14 | 0.12 | 7.04 | -0.24 – 2.06 | 0.29 | **0.001** |
| **Statins** | 0.62 | -.037 – 1.60 | 0.12 | 0.20 | 4.15 | 0.55 – 7.74 | 0.18 | **0.033** |
| **EAT(cm²)** | 0.17 | 0.07 – 0.28 | 0.28 | **0.002** | 0.60 | 0.22 – 0.97 | 0.33 | **0.002** |

*PWV* pulse wave velocity, *LVMI* left ventricular mass index, *BMI* body mass index, *BP* blood pressure, *CAD* coronary artery disease, *LEAD* lower extremity arterial disease, *ACE* angiotensin converting enzyme, *EAT* epicardial adipose tissue

Table S3. Multivariable Associations with LV Mass Index

|  | **Clinical** | | | | **Clinical + EAT** | | | |
| --- | --- | --- | --- | --- | --- | --- | --- | --- |
|  | **β** | **95% CI** | **β (S)** | **p value** | **β** | **CI 95%** | **β (S)** | **p value** |
| **Male** | -10.99 | -14.06 - -7.93 | -0.52 | **<0.001** | -10.73 | -13.67- -7.80 | -0.51 | **<0.001** |
| **Hypertension** | 4.88 | 1.72 – 8.04 | 0.22 | **0.003** | 4.44 | 1.41-7.46 | 0.21 | **0.004** |
| **EAT** |  |  |  | | 0.16 | -0.17 – 0.49 | 0.07 | 0.34 |

*EAT* epicardial adipose tissue, *BMI* body mass index, *CAD* coronary artery disease, *LEAD* lower extremity arterial disease

Figure S1. Correlations between EAT and IL-6 (A) and CD40L (B).


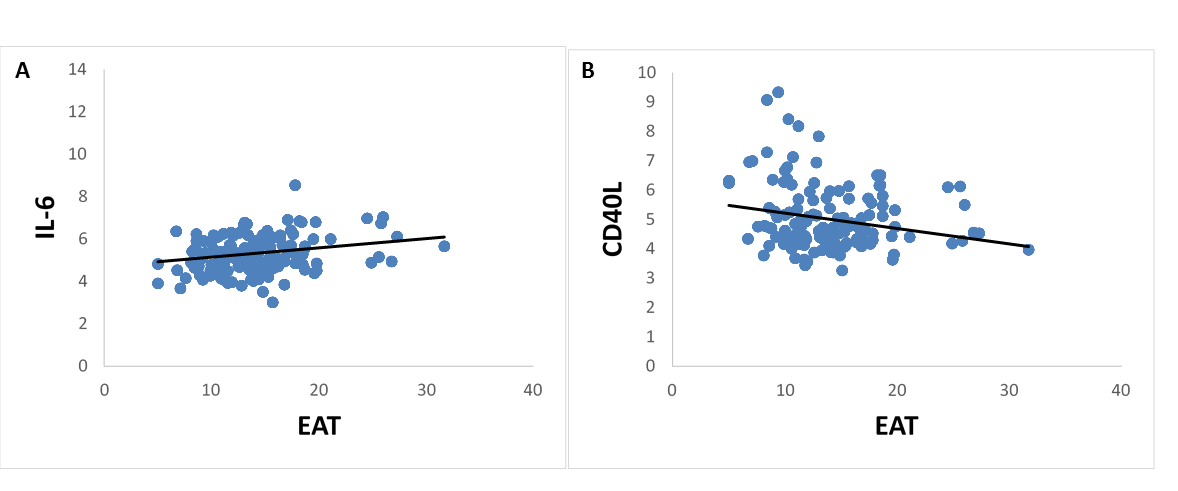

Supplement: Supplementary file 1 — Table S1. Correlations between cardiovascular biomarkers and epicardial adipose tissue (EAT), pulse wave velocity (PWV) and left ventricular mass index (LVMI). Table S2. Univariable Linear Regression of Clinical Variables and EAT with Pulse Wave Velocity and LV Mass Index. Table S3. Multivariable Associations with LV Mass Index. Figure S1. Correlations between EAT, IL-6 and CD40L. (DOCX 88 kb) [file 12872_2018_770_MOESM1_ESM.docx]
